# Regional high iron deposition is linked with cognitive impairments in peritoneal dialysis: a quantitative susceptibility mapping study

**DOI:** 10.3389/fnagi.2025.1660734

**Published:** 2025-09-24

**Authors:** Dashan Li, Yongjie Yin, Shan Jiang, Jiuyu Yin, Luyao Yu, Nan Feng, Zhaohua Sun, Zilian Chen, Hongting Xu, Yu Zhou, Jie Fang, Xiangming Qi, Haibao Wang, Yonggui Wu

**Affiliations:** ^1^Department of Nephropathy, The First Affiliated Hospital of Anhui Medical University, Hefei, Anhui, China; ^2^Department of Radiology, The First Affiliated Hospital of Anhui Medical University, Hefei, Anhui, China

**Keywords:** peritoneal dialysis, serum ferritin, iron deposition, cognitive impairment, quantitative susceptibility mapping

## Abstract

**Objective:**

Peritoneal dialysis (PD) patients demonstrate distinct iron homeostasis imbalances. However, the relationship between brain iron and cognitive impairment in this population remains poorly elucidated.

**Methods:**

This study enrolled 52 PD patients and 49 healthy controls (HCs). Quantitative susceptibility mapping (QSM) was employed to quantify cerebral iron deposition. Cognitive function was assessed using the Montreal Cognitive Assessment (MoCA) and a comprehensive neuropsychological test battery. Dose–response relationships between iron metabolism parameters and cognitive performance were analyzed using generalized additive models (GAMs).

**Results:**

PD patients exhibited significantly higher iron deposition in the left amygdala and right putamen compared to HCs. Serum ferritin (SF) demonstrated an approximately inverted U-shaped relationship with MoCA scores, with an inflection point at 258.4 μg/L (*p* < 0.001). Every 100 μg/L increase in SF beyond this threshold was associated with a 3.1-point decrease in MoCA score. Iron deposition in the left amygdala showed significant correlations with scores on the Digit Symbol Test (DST), Self-Rating Depression Scale (SDS), Self-Rating Anxiety Scale (SAS), and Verbal Fluency Test (VFT), but exhibited no direct association with peripheral iron metabolism parameters.

**Conclusion:**

In peritoneal dialysis patients, abnormal cerebral iron deposition predominantly localizes to limbic-basal ganglia regions. Iron accumulation in the left amygdala may specifically mediate the development of multi-domain cognitive impairment. QSM represents a sensitive technique for early detection of pathological iron accumulation.

## 1 Introduction

Patients with chronic kidney disease (CKD) exhibit a significantly higher prevalence of cognitive impairment compared to the general population, with underlying mechanisms involving multifactorial interactions such as uremic toxin accumulation, inflammatory responses, oxidative stress, and cerebrovascular lesions (Drew et al., 2019; Viggiano et al., 2020). As a renal replacement therapy for end-stage renal disease (ESRD), peritoneal dialysis (PD) partially clears metabolic waste products; however, the incidence of cognitive impairment in PD patients remains notably elevated (22.3–74.5%), severely compromising treatment adherence and quality of life (Kalirao et al., 2011; Liu et al., 2015; Shea et al., 2016). In recent years, the role of iron metabolism dysregulation in neurodegenerative disorders has garnered increasing attention (Wu et al., 2023).

Iron is an essential element for neurotransmitter synthesis and myelination, and its metabolic imbalance can induce oxidative stress damage, leading to neuronal apoptosis and synaptic dysfunction (Munoz and Humeres, 2012). Serum ferritin (SF) is a reliable marker for assessing peripheral iron stores. Notably, iron metabolism disorders exhibit a dose-dependent impact on cognition—moderate iron levels sustain neuronal plasticity, whereas overload triggers neuroinflammatory cascades through microglial activation (Kim et al., 2023; Liu et al., 2025; Nunez and Hidalgo, 2019). Compared to hemodialysis (HD) patients, PD patients demonstrate distinct iron dysregulation patterns: (1) Chronic peritoneal exposure to high-glucose dialysate induces local oxidative stress and upregulates hepcidin expression, leading to intestinal iron absorption inhibition and reticuloendothelial iron sequestration (Yamaji et al., 2004). PD-associated protein loss (particularly transferrin) exacerbates functional iron deficiency, necessitating frequent intravenous iron supplementation that perpetuates an “iron overload-inflammation-hepcidin elevation” vicious cycle (Niikura et al., 2019; Zou et al., 2021); (2) Residual renal function differences influence iron regulation: Compared to HD counterparts, PD patients retain stronger residual glomerular filtration function during the early-to-middle stages of treatment (Moist et al., 2000; Sinnakirouchenan and Holley, 2011), which theoretically supports their greater urinary iron excretion capacity. However, this regulatory effect on systemic iron load may be counterbalanced by peritoneal iron loss (Niikura et al., 2019). This unique iron homeostasis imbalance potentially facilitates cerebral iron accumulation through blood–brain barrier permeability alterations, a phenomenon that has been overlooked in existing research. Nevertheless, current studies reveal significant discrepancies regarding iron metabolism-cognitive impairment associations: In Alzheimer’s disease populations, elevated SF levels show significant negative correlation with MMSE score decline (Li et al., 2024). Conversely, higher SF values were found to be associated with higher cognitive function in infants aged 1–3 years (Parkin et al., 2020). Therefore, the role of iron metabolism in cognitive impairment remains to be explored.

The advent of Quantitative Susceptibility Mapping (QSM) technology has revolutionized research on cerebral iron deposition. Compared to conventional T2-weighted imaging, QSM leverages phase information to quantitatively resolve tissue susceptibility differences, enabling precise discrimination between paramagnetic substances such as iron and calcium, with a spatial resolution of 1 mm^3^ (Liu et al., 2015; Wang et al., 2022). This technique significantly enhanced sensitivity in detecting iron deposition within deep gray matter nuclei (e.g., basal ganglia and hippocampus)(Ning et al., 2019). Studies have established significant correlations between iron accumulation in regions such as the putamen and caudate nucleus and impairments in executive function and memory (Modica et al., 2015; Zhi et al., 2024). Moreover, the high sensitivity of QSM enables the detection of early subclinical iron deposition changes, offering a predictive biomarker for cognitive decline. For instance, in patients with CKD stages 1–4, increased susceptibility values in the putamen have been observed to precede the onset of abnormalities in conventional neuropsychological tests (Wang et al., 2023). This temporal precedence highlights QSM’s potential as a prospective tool for stratifying cognitive risk in PD patients, facilitating early intervention before overt neuropsychological deficits emerge. However, a critical knowledge gap persists in PD research, where the absence of QSM-based investigations severely hinders the development of personalized iron management strategies for PD populations.

Although existing studies have revealed the role of iron metabolism dysregulation in CKD-related cognitive impairment, critical gaps persist in research targeting PD populations: (1) Most investigations focus on HD patients, overlooking the unique iron homeostasis alterations in PD patients caused by peritoneal glucose exposure and albumin loss; (2) The association between cerebral iron deposition and domain-specific cognitive deficits (such as working memory and processing speed) in PD patients remains incompletely understood. (3) Clinical observations have revealed that PD patients commonly exhibit dysregulation of iron metabolism. Long-term peritoneal dialysis may contribute to systemic iron overload, while the association between peripheral blood levels of ferritin and cerebral iron deposition remains inconclusive.

Here, we performed QSM analyses to compare the magnetic susceptibility of the regional brain between patients with PD and healthy controls (HC). By integrating neuropsychological assessments, we systematically investigate the associations among iron metabolism index, cerebral iron deposition, and cognitive impairment in PD patients. This study combined iron metabolism, QSM, and neuropsychological data to elucidate the neuropathological mechanisms of systemic iron overload and central nervous system deposition in cognitive impairment.

## 2 Materials and methods

### 2.1 Study population

This cross-sectional study was approved by the Research Ethics Committee of the First Affiliated Hospital, Anhui Medical University (Ethics approval number: PJ2024-06-63). All participants involved in this study provided written informed consent in accordance with the Declaration of Helsinki.

From June 2024 to February 2025, patients who underwent clinical evaluation, laboratory tests, and multimodal neuroimaging assessments at the Department of Nephropathy of The First Affiliated Hospital of Anhui Medical University were enrolled in this study. Furthermore, Fifty-two HCs matched for gender, age, and years of education were recruited from the local community, with three participants excluded due to cerebral infarction or claustrophobia. All patients fulfilled the following inclusion criteria: aged 18–80 years, undergoing continuous ambulatory peritoneal dialysis (CAPD) with standard lactate-based peritoneal dialysis solution (containing 15 or 25 g glucose, 5.38 g sodium chloride, calcium chloride, 0.051 g magnesium chloride, and 4.48 g sodium lactate per 1,000 mL), stable dialysis duration >3 months, and right-handedness. Exclusion criteria were as follows: (1) History of craniocerebral injury or other neurological disorders (e.g., traumatic brain injury, intracranial space-occupying lesions); (2) Prior history of impaired consciousness, affective disorders, or neuropsychological conditions (e.g., depression, anxiety disorders, schizophrenia); (3) Documented substance dependence or abuse (including alcohol, medications, or illicit drugs); (4) Patients with renal transplantation, acute infections, heart failure, or severe hepatic dysfunction; (5) Inability to complete neuropsychological assessments or cranial magnetic resonance imaging (MRI). All imaging data were independently evaluated by two experienced radiologists blinded to participants’ clinical and cognitive profiles. Participants exhibiting suboptimal image quality, severe white matter disease, cerebral infarction (excluding lacunar infarcts), or intracranial hemorrhage were excluded from subsequent analyses.

### 2.2 Clinical and biochemical assessments

Clinical and laboratory data of patients were obtained using standardized forms. The following indicators were extracted: Demographic data, including age, gender, years of education, and dialysis duration; Physical examination variables, including systolic blood pressure (SBP), diastolic blood pressure (DBP), height, and body weight with dry abdomen; Health history, including the history of diabetes and peritonitis, which was collected from the electronic medical records of our hospital; and laboratory variables from serum, urine, and peritoneal dialysis fluid, including white blood cell count, hemoglobin, serum albumin, blood urea nitrogen (BUN), creatinine, uric acid, residual glomerular filtration rate (rGFR), C-reactive protein (CRP), serum iron metabolism indicators, alkaline phosphatase, total cholesterol, high-density lipoprotein cholesterol (HDL-C), low-density lipoprotein cholesterol (LDL-C), triglycerides, calcium-phosphorus product, serum bicarbonate concentration, intact parathyroid hormone (iPTH), etc. Standard laboratory methods were used to measure the dialysate/plasma creatinine ratio (D/P Cr), total creatinine clearance (CCr), and total Kt/V on an automated chemical analyzer. The specific details are shown in Table 1. Residual renal function, expressed as rGFR, was calculated using the following formula:rGFR = 1/2[urine Cr(μmol/L)/serum Cr(μmol/L) + urine urea(mmol/L)/serum urea(mmol/L)] × urine volume(mL)/1,440 (Nolph et al., 1993). These values were obtained through the peritoneal equilibrium test (PET). Patients should receive standard CAPD treatment before the PET, and the nocturnal peritoneal dialysis fluid should be retained in the abdominal cavity for 8–12 h. No blood biochemical tests were performed on the control subjects.

**Table 1:** Demographical and clinical biochemical data for the peritoneal dialysis patients included in the study.

Variables	All (*n* = 52)	PD-MCI (*n* = 34)	PD-CN (*n* = 18)	t/χ^2^/z	*P* value
Age (years)	53 (47, 57)	54 (50, 57)	48 (42, 56)	−2.265	0.023
Female [*n* (%)]	33 (63.4)	21 (61.8)	12 (66.7)	0.122	0.727
Education (years)	8 (6, 9)	7 (6, 8)	10 (8, 12)	−3.895	<0.001
Dialysis period (months)	23 (12, 61)	27 (13, 69)	20 (12, 52)	−1.059	0.290
BMI (kg/m^2^)	23.17 ± 2.91	23.52 ± 2.84	22.51 ± 3.02	−1.185	0.242
Diabetes [n (%)]	11 (21.1)	8 (23.5)	3 (16.7)	-	0.727
Incidence of peritonitis [n (%)]	14 (26.9)	7 (20.6)	7 (38.9)	-	0.197
Systolic blood pressure (mmHg)	142.83 ± 17.59	142.12 ± 18.96	144.17 ± 15.11	0.396	0.694
Diastolic blood pressure (mmHg)	91.35 ± 12.17	88.82 ± 12.08	96.11 ± 11.15	2.124	0.039
Laboratory data					
White blood cell count (10^9^/L)	6.74 ± 2.29	7.04 ± 2.19	6.17 ± 2.43	−1.311	0.196
Hemoglobin (g/L)	100.79 ± 17.80	101.21 ± 19.25	100.00 ± 15.17	−0.230	0.819
Albumin (g/L)	37.85 ± 3.35	38.13 ± 3.50	37.33 ± 3.07	−0.810	0.422
Alkaline phosphatase (U/L)	83.35 (63.25, 105.93)	84.7 (65.5, 102.0)	77.3 (62.4, 123.1)	−0.115	0.908
LDH (U/L)	229.25 (193.08, 264.35)	236.0 (205.4, 266.9)	210.85 (187.3, 251.3)	−1.135	0.256
BUN (mmol/L)	21.39 (17.19, 24.01)	21.39 (17.57, 24.29)	21.18 (16.48, 23.28)	−0.529	0.597
Uric acid (μmol/L)	434.48 ± 80.79	450.20 ± 86.43	404.78 ± 60.41	−1.984	0.053
Serum creatinine (umol/L)	968.31 ± 260.30	949.06 ± 282.34	1004.66 ± 215.37	0.729	0.469
rGFR ml/min/1.73m^2^	1.37 (0, 5.10)	2.24 (0.00, 5.62)	0.57 (0.00, 3.28)	−1.188	0.235
Total cholesterol (mmol/L)	4.56 ± 0.99	4.51 ± 1.11	4.66 ± 0.74	0.522	0.604
Triglyceride (mmol/L)	1.58 (1.07, 2.79)	1.67 (1.06, 3.54)	1.42 (1.08, 2.66)	−0.616	0.538
LDL-C (mmol/L)	2.99 ± 0.72	2.92 ± 0.75	3.14 ± 0.66	1.029	0.308
HDL-C (mmol/L)	1.17 ± 0.30	1.16 ± 0.33	1.18 ± 0.24	0.238	0.813
iPTH (pg/ml)	258 (101, 396)	316.72 ± 211.71	271.98 ± 278.73	−0.649	0.520
Calcium-phosphorus product	3.85 ± 0.95	4.03 ± 0.89	3.53 ± 1.01	−1.848	0.071
Bicarbonate (mmol/L)	24.96 ± 2.62	24.97 ± 2.68	24.94 ± 2.57	−0.037	0.970
Serum iron (umol/L)	11.04 ± 4.16	10.68 ± 4.11	11.71 ± 4.27	0.849	0.400
Serum ferritin (ug/L)	159.75 (98.68, 327.38)	337.05 (281.60, 440.20)	257.05 (243.10, 271.90)	−3.789	<0.001
Transferrin(g/L)	1.82 (1.66, 2.04)	1.84 (1.67, 2.21)	1.81 (1.63, 1.88)	−1.000	0.317
Transferrin saturation (%)	22.28 ± 11.51	30.01 ± 6.64	23.52 ± 4.39	−3.728	<0.001
TIBC (umol/L)	41.01 (38.33, 46.71)	41.85 (38.49, 48.99)	40.55 (36.82, 43.11)	−1.164	0.245
CRP (mg/L)	1.79 (0.54, 5.68)	2.17 (1.00, 5.61)	0.87 (0.43, 5.70)	−1.173	0.241
D/P Cr at 4 h	0.62 ± 0.11	0.60 ± 0.08	0.66 ± 0.13	1.843	0.071
Total CCr (L/week)	50.95 (45.31, 59.49)	50.71 (45.61, 55.41)	52.06 (39.57, 63.83)	−0.115	0.908
Total Kt/V	1.94 (1.43, 2.16)	1.95 (1.38, 2.16)	1.94 (1.50, 2.23)	−0.144	0.885
Peritoneal function				1.728	0.189
High/high average transport (n (%))	17 (32.7)	9 (26.5)	8 (44.4)		
Low/low average transport (n (%))	35 (67.3)	25 (73.5)	10 (55.6)		

Values are presented as means ± SD or interquartile range or percentages. PD-MCI, peritoneal dialysis with mild cognitive impairment; PD-CN, peritoneal dialysis with normal cognition; BMI, body mass index; LDH, lactate dehydrogenase; BUN, blood urea nitrogen; rGFR, residual glomerular filtration rate; LDL-C, low-density lipoprotein cholesterol; HDL-C, high-density lipoprotein cholesterol; TIBC, total iron binding capacity; CRP, c-reactive protein; iPTH, intact parathyroid hormone; D/P Cr, dialysate/plasma creatinine ratio; CCr, creatinine clearance rete; Kt/V, urea clearance index.

### 2.3 Cognitive and behavioral assessments

Neurological examinations were conducted for all participants by two senior investigators in a quiet environment one day prior to MRI examinations. Cognitive function was evaluated using the Beijing-Chinese version of the MoCA, a validated cognitive screening instrument, as the primary outcome measure (Lu et al., 2011). It assesses seven domains: visuospatial/executive function, naming, attention, abstraction, language, delayed memory, and orientation, with a total score ranging from 0 to 30 points (the higher the score, the better the function). To mitigate educational bias, an additional point was added to the final score for individuals with educational attainment <12 years. All participants of mild cognitive impairment (MCI) were diagnosed in adherence to the Petersen criteria, which encompass: (1) subjective memory complaints, (2) MoCA score <26 (adjusted for educational attainment if necessary), (3) normal function in daily life, and (4) absence of dementia (Marcos et al., 2016). In addition, A standardized neuropsychological battery was administered to evaluate emotional status, cognitive processing speed, attentional capacity, executive function, and language abilities: (1) Emotional profiling: self-rating anxiety scale (SAS) and self-rating depression scale (SDS) were utilized to quantify anxiety and depressive symptoms; (2) Processing speed: symbol digit modalities test (SDMT) assessed visual-motor information processing efficiency. The outcome measure is the number of correct symbol-digit pairings completed within a fixed time period. (3) Attention metrics: digit span test (forward and backward versions) measured auditory-verbal working memory and attentional control. (4) Executive function: trail making test (TMT) evaluated cognitive flexibility, while stroop color-word test quantified inhibitory control. The core outcome measure of the former is the total time (in seconds) required to complete the trail-making task, while the outcome indicator of the latter is the total time (in seconds) needed to finish all items under each condition. (5)Language competence: verbal fluency test (VFT)-Fruit Category was employed to assess semantic retrieval and lexical access.

### 2.4 Imaging protocol

MRI images of each subject were acquired on a 3.0 T MRI system (Siemens Healthcare, Erlangen, Germany) equipped with a 64 - channel head matrix coil. All participants were instructed to maintain strict supine positioning throughout scanning procedures, with foam padding and noise-reducing earplugs applied to minimize motion artifacts and acoustic interference. Quantitative susceptibility mapping was obtained using a three-dimensional multi-echo gradient echo sequence with the following parameters: echo time (TE) 6.3 ms, repetition time (TR) 35 ms, field of view (FOV) 220 × 192 mm^2^, flip angle 20°, acquisition matrix 416 × 291, slice thickness 2.0 mm with 0.4 mm interslice gap, 72 contiguous slices, and total acquisition time 5.3 min. Structural imaging employed a T1-weighted 3D brain volume sequence (MPRAGE) with optimized parameters: TE 2.9 ms, TR 2300 ms, FOV 256 × 240 mm^2^, flip angle 9°, isotropic matrix 256 × 256, 1.0 mm slice thickness with 0.5 mm gap, 208 slices, achieving full brain coverage in 5.1 min. Additional FLAIR imaging was acquired using the following protocol: TE 98 ms, TR 8000 ms, FOV 220 × 200 mm^2^, flip angle 150°, matrix size 320 × 224, 5.0 mm slice thickness with 1.0 mm gap, 24 slices, completed within 98 s.

### 2.5 Quantitative susceptibility mapping processing and standardization protocol

The QSM data were reconstructed and analyzed using the SuscEptibility mapping PIpeline toolfor phAse images (SEPIA) toolbox (Chan and Marques, 2021) in the MATLAB program (MathWorks, Natick, MA). First, non-brain tissues were removed from the raw phase and magnitude images using the Brain Extraction Tool (BET) with a fractional intensity threshold of 0.3. Phase unwrapping was subsequently performed using a Laplacian-based algorithm to resolve phase discontinuities. Local background magnetic field contributions were corrected via the Laplacian Boundary Value (LBV) method. The susceptibility distribution map was reconstructed using an inverse filter with a k-space threshold of 0.15 to suppress high-frequency noise artifacts. Spatial smoothing was applied using a Gaussian kernel with a full width at half maximum (FWHM) of 2 mm to enhance signal-to-noise ratio (SNR).

**Figure 1:**
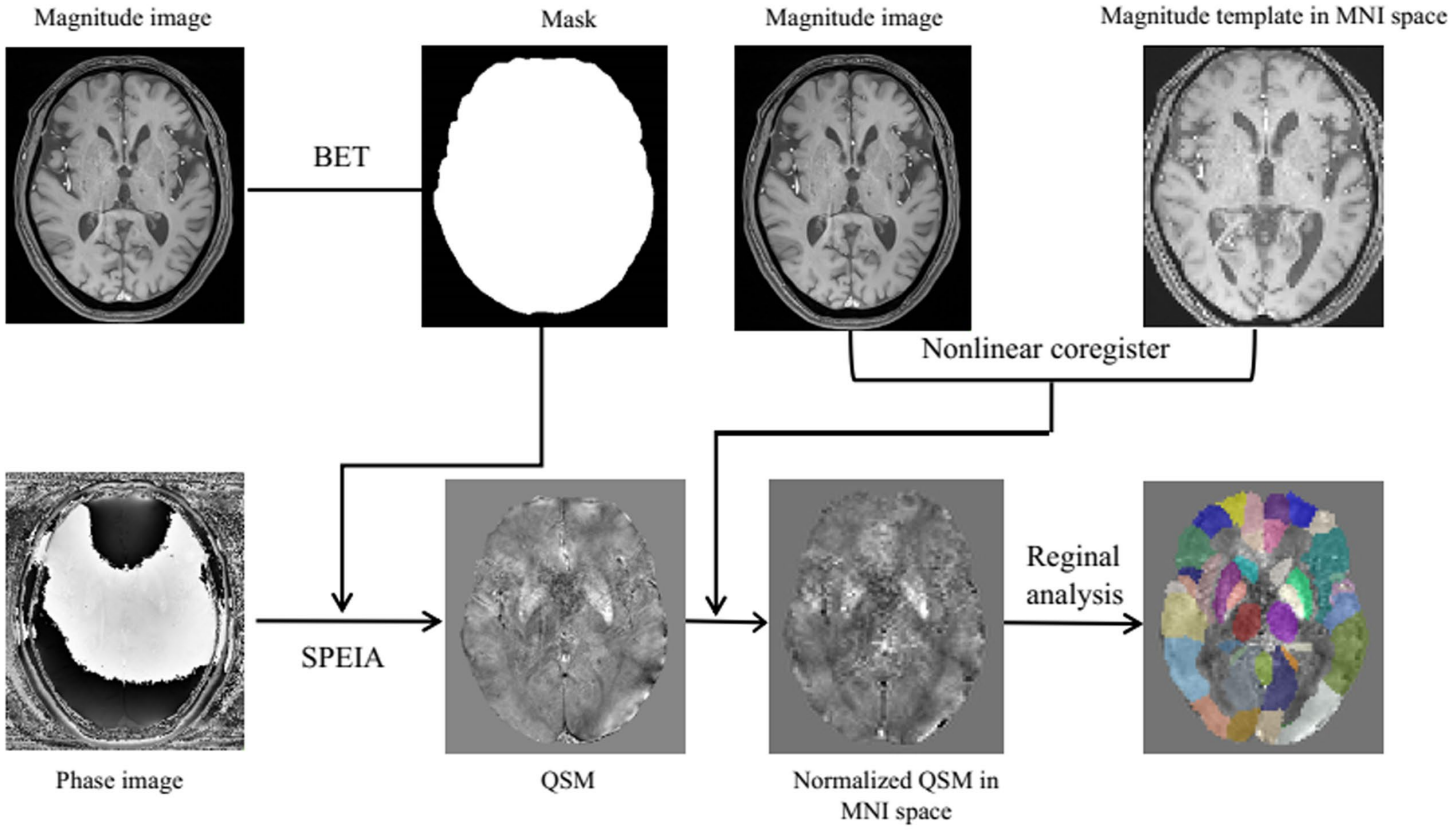
Flowchart of QSM image reconstruction and spatial normalization processing. BET, brain extraction tool; MNI, montreal neurological institute; SEPIA, SuscEptibility mapping PIpeline toolfor phAse images; QSM, quantitative susceptibility mapping.

The subsequent processing steps were performed using SPM12 based on MATLAB 2017a. The reconstructed QSM images were spatially normalized to the Montreal Neurological Institute (MNI) standard space through a one-step registration approach. The specific workflow consisted of two phases: (1) The Normalise (Estimate) module in SPM12 was employed to perform non-linear registration between the magnitude map of the first echo sequence from individual subjects and the tissue probability map (TPM) in MNI space, during which the deformation matrix was generated and preserved; (2) The Normalize (Write) module was then applied to propagate the preserved deformation field onto the QSM images, achieving spatial normalization of QSM data into MNI space (Figure 1). Finally, all normalized QSM images were resliced to achieve isotropic spatial resolution of 2 × 2 × 2 mm^3^ through voxel interpolation.

The normalized susceptibility maps were aligned to the automated anatomical Labeling (AAL) parcellation template, and voxel-wise magnetic susceptibility values within each of the 116 brain regions were automatically extracted using code written in MATLAB R2017a. Regional susceptibility characteristics were quantified as the arithmetic mean of voxel values per ROI. To evaluate the relationship between magnetic susceptibility values and cognitive performance, we selected iron-rich subcortical nuclei as ROIs, guided by prior research findings and accounting for the anatomical limitations of the AAL template (Haacke et al., 2005; Moon et al., 2016). The ROIs in this study included the thalamus, caudate nucleus, globus pallidus, putamen, hippocampus, and amygdala.

### 2.6 Statistical analysis

All statistical analyses were performed using R (version 4.3.1; R Foundation for Statistical Computing, Vienna, Austria; https://www.R-project.org), EmpowerStats (version 3.0; X&Y Solutions Inc., Boston, MA; https://www.empowerstats.com), and SPSS software (version 25.0; IBM Corp., Armonk, NY, United States). Normality of data distribution was assessed using the Kolmogorov–Smirnov test. Continuous variables conforming to a normal distribution were expressed as mean ± standard deviation (SD), whereas skewed variables were reported as median with interquartile range (IQR). Categorical variables were summarized as frequencies and percentages.

For group comparisons, independent two-sample t-tests and Mann–Whitney U tests were applied to evaluate differences in normally and non-normally distributed continuous variables, respectively, between the PD and HC groups. Pearson’s chi-square test was utilized for categorical variables. For multiple comparisons, tests were corrected using the Bonferroni method (*p* < 0.05/116 = 0.0004).

Multivariable linear regression models were constructed to examine the association between MoCA scores and ferritin levels. Generalized additive models (GAM) with smooth curve fitting were employed to explore potential curve relationships between MoCA scores and ferritin. To investigate threshold effects in the MoCA-ferritin correlation, subgroup analyses were conducted using stratified multivariable regression models, incorporating interaction terms to assess variability across predefined subgroups (e.g., age, gender, or BMI). The correlation was analyzed using Pearson’s correlation coefficient for normally distributed data or Spearman’s rank correlation coefficient for non-normally distributed data between the mean QSM values of ROIs, cognitive scores, and laboratory data. However, to mitigate the elevated risk of type I errors arising from multiple comparisons, false discovery rate (FDR) correction methodology was implemented throughout the analytical process. The statistically significant *p* value was set at 0.05.

## 3 Results

### 3.1 Demographics and clinical characteristics

This study enrolled 52 patients undergoing peritoneal dialysis, categorized into peritoneal dialysis with mild cognitive impairment (PD-MCI) (*n* = 34) and peritoneal dialysis with normal cognition (PD-CN) (*n* = 18) groups based on cognitive function assessments. Demographic and clinical characteristics are summarized in Tables 1, 2. Significant intergroup differences were observed in demographic profiles: the PD-MCI group exhibited a higher median age (*p* = 0.023) and shorter educational attainment (median 7 years vs. 10 years, *p* < 0.001). Gender distribution (61.8% females in PD-MCI vs. 66.7% in PD-CN) and other baseline characteristics, including BMI, diabetes prevalence, and dialysis duration, showed no significant differences (all *p* > 0.05).

**Table 2:** Neuropsychological data for the peritoneal dialysis patients and healthy controls included in the study.

Variables	PD (*n* = 52)	HC (*n* = 49)	*P* value
Age (years)	50.83 ± 7.90	49.37 ± 8.12	0.359
Female [*n* (%)]	34 (64.2)	34 (69.4)	0.575
Education (years)	8 (6, 9)	9 (8, 9)	0.224
SAS (score)	38.75 (32.50, 41.25)	27.50 (26.25, 31.25)	<0.001
SDS (score)	42.50 (37.50, 51.25)	27.50 (25.00, 32.50)	<0.001
Cognitive screening			
MoCA (score)	23 (17, 27)	27 (25, 28)	<0.001
SDMT (score)	21.55 ± 15.54	39.49 ± 15.45	<0.001
DST-forwards (score)	6 (5, 8)	9 (9, 10)	<0.001
DST-backwards (score)	4 (3, 5)	6 (5, 7)	<0.001
Stroop-word (s)	33.16 (23.65, 43.67)	26.96 (22.19, 32.51)	0.006
Stroop-color (s)	47.74 (37.57, 66.01)	39.17 (34.86, 46.07)	0.004
Stroop-interference (s)	80.13 (65.00, 106.89)	66.00 (58.75, 81.00)	0.003
TMT-A (s)	68.59 (49.10, 82.38)	62.00 (45.00, 84.00)	0.547
TMT-B (s)	83.82 (55.02, 100.48)	84.00 (67.00, 131.00)	0.127
VFT (score)	10 (8, 12)	12 (10, 15)	0.001

Values are presented as means ± SD or interquartile range or percentages. PD, peritoneal dialysis; HC: healthy control; SAS, self-rating anxiety scale; SDS, self-rating depression scale; MoCA, montreal cognitive assessment; SDMT, symbol digit modalities test; DST, digit span test; TMT, trail making test; VFT, verbal fluency test.

Laboratory analyses revealed marked disparities in iron metabolism parameters: serum ferritin levels were significantly elevated in the PD-MCI group (*p* < 0.001), with correspondingly higher transferrin saturation (*p* < 0.001). Hemodynamically, the PD-CN group demonstrated higher diastolic blood pressure (*p* = 0.039). Although the PD-MCI group showed a trend toward higher uric acid levels and calcium-phosphorus product values, these differences approached but did not reach statistical significance (*p* = 0.053 and *p* = 0.071, respectively). No significant intergroup variations were detected in the remaining laboratory and clinical parameters, including inflammatory markers (CRP), peritoneal transport characteristics, and nutritional indices (all *p* > 0.05).

As shown in Table 2, patients undergoing PD exhibited multidimensional neuropsychological impairments compared to HC. The two groups showed no statistically significant differences in age, gender distribution, or years of education (all *p* > 0.05). However, neuropsychological assessments revealed the following: (1) Emotional dimensions: The PD group scored significantly higher on the SAS and SDS than the HC group (both *p* < 0.001). (2) Global cognitive function: The PD group demonstrated significantly lower MoCA scores (*p* < 0.001). (3) Processing speed: The SDMT scores were markedly reduced in the PD group (*p* < 0.001). (4) Working memory: Both forward and backward Digit Span Test scores were significantly diminished in the PD group (both *p* < 0.001). (5). Executive function: The Stroop Test revealed significantly prolonged completion times for word naming, color naming, and interference tasks in the PD group. (6). Verbal fluency: The PD group showed significantly lower scores on the VFT. Notably, no significant differences were observed between the two groups in completion times for TMT-A or TMT-B. These findings suggest that PD patients experience broad cognitive impairment, with pronounced deficits in information processing speed, working memory, executive function, and emotional regulation, while visual search and task-switching abilities remain relatively preserved.

### 3.2 Association between SF and cognitive function

This study employed a two-stage regression analysis to investigate factors influencing MoCA scores in peritoneal dialysis patients. Univariate linear regression analysis revealed significant associations between MoCA scores and age (*β* = −0.348, 95% CI: −0.554 to −0.143, *p* = 0.001), years of education (*β* = 1.191, 95% CI: 0.485 to 1.896, p = 0.001), serum ferritin (*β* = −0.040, 95% CI: −0.054 to −0.025, *p* < 0.001), and transferrin saturation (*β* = −0.310, 95% CI: −0.567 to −0.053, *p* = 0.019) (Supplementary Table 1). The borderline significant variable, dialysis duration (*p* = 0.052), was included in the multivariate model for adjustment. Following multivariate linear regression analysis, age (*β* = −0.204, 95% CI: −0.400 to −0.007, *p* = 0.042) and serum ferritin (*β* = −0.031, 95% CI: −0.046 to −0.017, *p* < 0.001) retained independent statistical significance (Table 3). Specifically, a 100 μg/L increase in serum ferritin correlated with a 3.1-point decline in MoCA scores. However, the associations with education years (*p* = 0.614) and transferrin saturation (*p* = 0.512) were attenuated in the adjusted model. These findings indicate that advanced age and iron overload are independent risk factors for cognitive impairment in peritoneal dialysis patients.

**Table 3:** Multivariable analysis of the relationship between MoCA scores and clinical indicators in peritoneal dialysis patients.

Variables	*β* (95%CI)	*P* value
Age (years)	−0.204 (−0.400, −0.007)	0.042
Education (years)	0.187 (−0.554, 0.928)	0.614
Dialysis period(months)	−0.028 (−0.069, 0.013)	0.181
Serum ferritin (ug/L)	−0.031 (−0.046, −0.017)	<0.001
Transferrin saturation (%)	−0.072 (−0.293, 0.148)	0.512

MoCA: montreal cognitive assessment.

**Figure 2:**
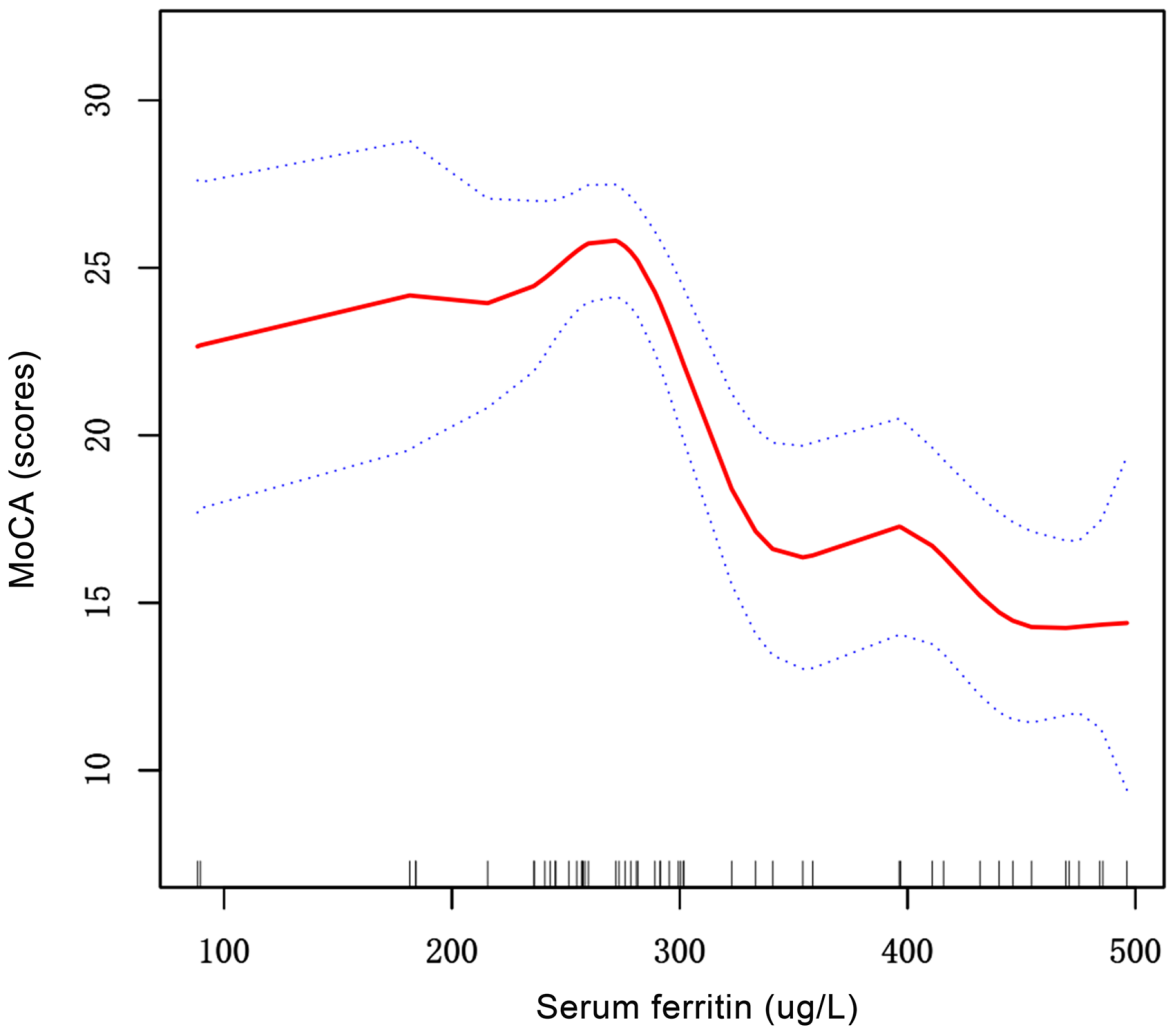
The correlation between serum ferritin and MoCA scores in patients with PD patients. The solid red line represents the smooth curve fit between variables. Blue bands represent the 95% of confidence interval from the fit. MoCA, montreal cognitive assessment; PD, peritoneal dialysis.

The generalized additive models with smooth curve fits were employed to delineate the nonlinear relationship between SF and MoCA scores, revealing a distinct inflection point at 258.4 μg/L (Figure 2). Subsequent threshold effect analysis using a two-piecewise linear regression model (Table 4) demonstrated divergent associations: above this threshold (SF ≥ 258.4 μg/L), each 1 μg/L increase in SF corresponded to a 0.053-point decline in MoCA scores (95% CI, −0.069 to −0.037). Conversely, below this threshold, each 1 μg/L increase in SF is linked to a slight increase of approximately 0.015 points in MoCA scores (95% CI, −0.018 to 0.047). Serum ferritin and MoCA scores appear to exhibit an inverted U-shaped relationship. Likelihood ratio tests confirmed significant improvement in model fit for the piecewise model compared to the standard linear model (*p* < 0.001), indicating superior data representation. The area between the upper and lower dashed lines is represented as 95% CI. Each point shows the magnitude of the SF and is connected to form a continuous line. Analyses were adjusted for age, educational attainment, dialysis duration, and transferrin saturation.

**Table 4:** Threshold effect analysis of serum ferritin on MoCA scores.

MoCA	*β* (95% CI), *P* value
Ferritin	
Model 1	
One line effect	−0.036 (−0.050, −0.022), <0.001
Model 2	
Turning point(K)	258.4
<258.4 slope1	0.015 (−0.018, 0.047), 0.374
>258.4 slope2	−0.053 (−0.069, −0.037), <0.001
slope2-1	−0.068 (−0.108, −0.028), 0.002
Model fit value at K	25.7 (24.0, 27.3)
Log-likelihood ratio test	<0.001

The model adjusted for age, education, dialysis period, and transferrin saturation. MoCA: montreal cognitive assessment.

Subgroup analyses were performed to assess the robustness and consistency of the SF-MoCA score relationship across potential effect modifiers, including age, gender, BMI, educational attainment, dialysis duration, diabetes, hypertension, and peritoneal transport status. Detailed results were reported in Supplementary Table 2. All interaction tests demonstrated non-significant effects (all *P* for interaction>0.05), indicating that the inverse association between SF and MoCA scores remained stable regardless of these stratification variables.

### 3.3 Increased regional brain iron in PD compared with controls

Following Bonferroni correction, significant intergroup differences in iron deposition were observed among PD and HC across specific brain regions (Table 5). The PD group exhibited significantly higher susceptibility values in the left amygdala, and right putamen compared to HC (*p* < 0.005/116 = 0.0004). No statistically significant differences were detected in other regions, including the hippocampus, caudate nucleus, globus pallidus, or thalamus (all *p* > 0.05/116 = 0.0004).

**Table 5:** Comparison of susceptibility (ppm) in selected structures among two groups.

ROI	HC (*n* = 49)	PD (*n* = 52)	Effect size (*d/Δ*)	*P* value*
Hippocampus_L	−0.0014 ± 0.009	−0.0021 ± 0.010	−0.074	0.713
Hippocampus_R	−0.0050 ± 0.008	−0.0046 ± 0.010	0.045	0.824
Amygdala_L	−0.0280 (−0.0465, −0.0164)	−0.0140 (−0.0236, 0.0022)	0.669	0.0002
Amygdala_R	−0.0267 (−0.0468, −0.0123)	−0.0153 (−0.0274, −0.0065)	0.379	0.017
Caudate_L	0.0071 ± 0.016	0.0066 ± 0.013	−0.031	0.878
Caudate_R	0.0141 (0.0068, 0.0293)	0.0120 (−0.0007, 0.0178)	−0.279	0.099
Putamen_L	0.0080 ± 0.019	0.0206 ± 0.017	0.689	0.001
Putamen_R	0.0124 ± 0.019	0.0268 ± 0.018	0.771	0.0002
Pallidum_L	0.0636 ± 0.032	0.0762 ± 0.038	0.358	0.076
Pallidum_R	0.0765 (0.0468, 0.0973)	0.0755 (0.0562, 0.0919)	0.165	0.625
Thalamus_L	−0.0071 (−0.0181, −0.0006)	−0.0022 (−0.0082, 0.0056)	0.552	0.006
Thalamus_R	−0.0079 ± 0.011	−0.0037 ± 0.010	0.381	0.059

Values are presented as means ± SD or interquartile range or percentages. *For the comparison among two groups (Bonferroni-corrected *P* < 0.05/116 = 0.0004). ROI: region of interest; PD: peritoneal dialysis; HC: healthy control.

### 3.4 Correlation analysis of regional brain iron with cognitive and iron metabolism indicators

**Figure 3:**
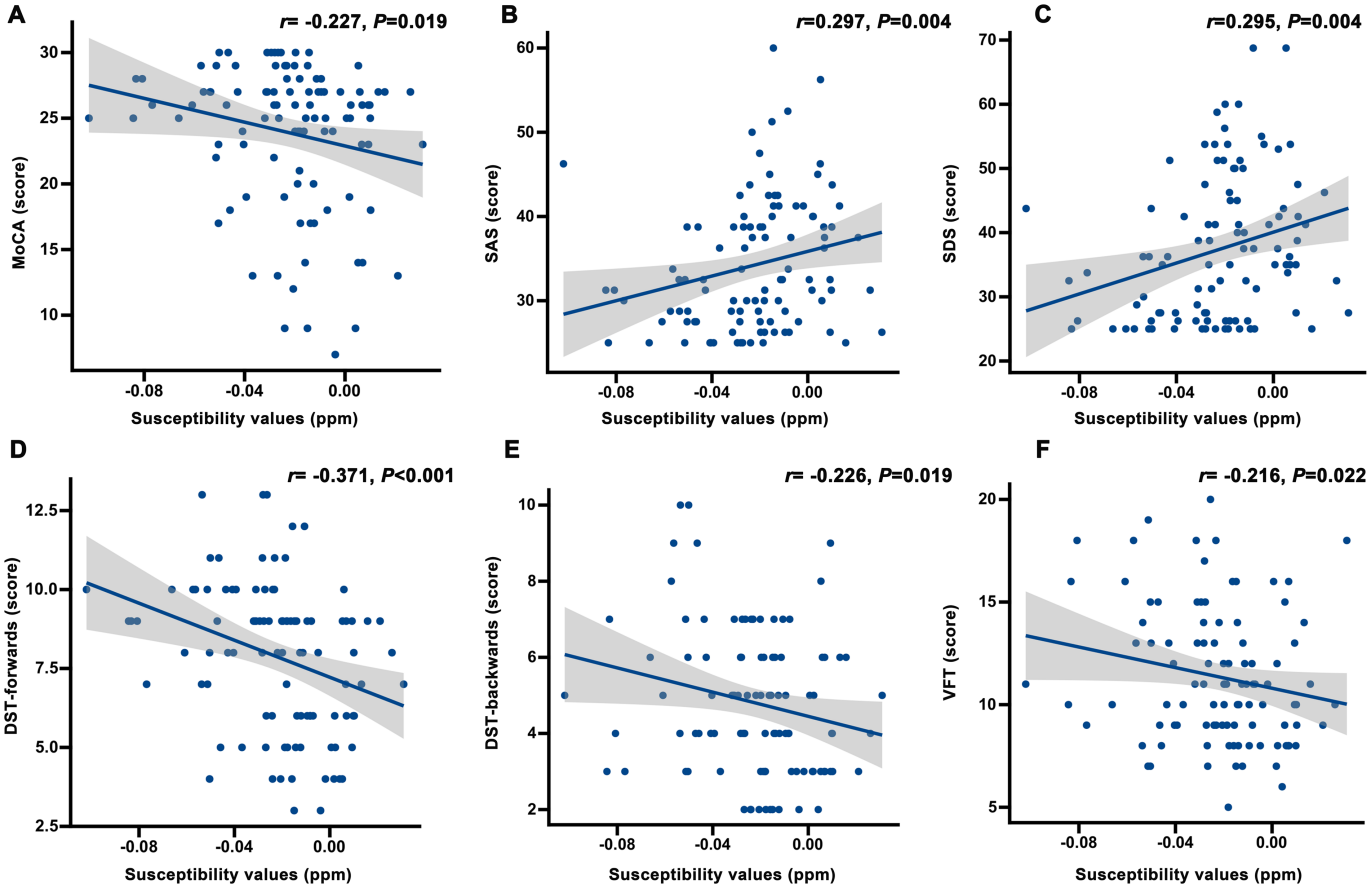
Correlation analysis between the susceptibility of left amygdala and neuropsychological data. **(A)** MoCA score; **(B)** SAS score; **(C)** SDS score; **(D)** DST-forwards score; **(E)** DST-backwards score; **(F)** VFT score. *p* values were corrected for false discovery rate. MoCA: montreal cognitive assessment; SAS: self-rating anxiety scale; SDS: self-rating depression scale; DST: digit span test; VFT: verbal fluency test.

The MoCA total score was significantly correlated with the susceptibility values of the left amygdala (Figure 3A). Subsequently, the correlation analysis revealed distinct correlation patterns between magnetic susceptibility values in the left amygdala and multidimensional cognitive assessments (Figures 3B–F and Supplementary Table 3). Specifically, the left amygdala exhibited significant positive correlations with SAS (r = 0.297, *p* = 0.004, FDR corrected) and SDS (r = 0.295, *p* = 0.004, FDR corrected), while demonstrating negative correlations with DST-forwards, DST-backwards, and VFT (r = −0.371, *p* < 0.001; r = −0.226, *p* = 0.019, and r = −0.216, *p* = 0.022, FDR corrected, respectively). However, the right putamen demonstrated no significant correlations with cognition function measures (all *p* > 0.05).

Moreover, the susceptibility values in the left amygdala showed no significant correlation with hemoglobin, serum iron, ferritin, or transferrin saturation in peritoneal dialysis patients (all *p* > 0.05) (Supplementary Figures 1A–D).

## 4 Discussion

This study systematically investigates, for the first time, the synergistic mechanism between iron metabolism abnormalities and region-specific cerebral iron deposition in cognitive impairment among peritoneal dialysis patients. Through multidimensional neuropsychological assessments combined with quantitative susceptibility mapping, two pivotal findings emerge: First, serum ferritin exhibits a inverted U-shaped relationship with global cognitive function, with its critical threshold effect (258.4 μg/L) establishing a basis for clinical iron overload management. Second, iron deposition in the left amygdala demonstrates specific associations with attention, emotional regulation, and language competence. These spatial distribution patterns contrast with Alzheimer’s disease-related pathological signatures (Zhi et al., 2024), suggesting a distinct neural regulatory mechanism underlying uremia-associated cognitive decline.

The present study revealed that 65% of PD patients exhibited cognitive impairment, a finding highly consistent with recent research by Bras et al. in European populations (Bras et al., 2024). However, a striking discrepancy was observed when compared with the 15% CI prevalence reported in Western populations by Noumann’s team (Neumann et al., 2018) which may be attributed to heterogeneity in assessment tools. Our multidimensional neuropsychological battery evaluation demonstrated significant impairments across multiple cognitive domains, particularly in executive function, processing speed, and language abilities. Notably, despite these pervasive cognitive deficits, PD therapy remains clinically feasible through its user-friendly home-based protocol (requiring only 3–4 daily exchanges), automated device support (e.g., APD cycler), and comprehensive medical team guidance (Mujais and Story, 2006). This suggests that PD implementation may have a relatively low cognitive threshold requirement for successful treatment adherence.

Iron, a pivotal regulator that mediates neurotoxic effects, has been closely associated with cognitive impairment through pathological iron accumulation. Studies demonstrate that iron overload induces neuronal damage via oxidative stress, inflammatory cascades, and ferroptosis pathways (Li et al., 2023; Xie et al., 2023). The heightened expression of transferrin receptor 1 (TfR1) within the central nervous system potentially facilitates iron ion transport across the blood–brain barrier (BBB), leading to cerebral iron deposition and subsequent neurodegenerative pathologies (Li et al., 2012; Zhang et al., 2021). Elevated serum ferritin levels exhibit significant correlations with cognitive impairment disorders including Alzheimer’s disease and cerebral hemorrhage, underscoring its neurotoxic potential (Lan et al., 2021; Li et al., 2024). Additionally, there is also evidence indicating that improvements in SF levels are associated with enhanced cognitive abilities, particularly in working memory, language processing, and executive functions (Raz et al., 2022; Rosell-Diaz et al., 2024). However, it is crucial to acknowledge that this association between peripheral ferritin and cognitive function stands in contrast to the broader body of research in Alzheimer’s disease. Most studies investigating Alzheimer’s disease have failed to detect consistent correlations between peripheral iron parameters (such as serum ferritin, transferrin saturation, and serum iron) and cognitive decline (Milward et al., 2010; Wang et al., 2025). Instead, compelling evidence in Alzheimer’s disease and other neurodegenerative disorders (e.g., Parkinson’s disease, vascular dementia) highlights that regional cerebral iron accumulation is the key pathological correlate of cognitive impairment (Moon et al., 2016; Guan et al., 2022). Current study only reveals a marked inverse correlation between serum ferritin concentrations and cognitive performance in peritoneal dialysis population. Multivariate regression analysis identifies both age and serum ferritin as independent risk factors for cognitive impairment. However, when using the Generalized Additive Model and curve fitting for curve analysis, an inverted U-shaped relationship was identified between serum ferritin levels and MoCA scores in peritoneal dialysis patients and the *β* value are low. Therefore, further research is necessary to determine the clinical significance of these findings. Mechanistically, iron accumulation may impair cognition through three synergistic pathways: (1) Iron-mediated free radical generation exacerbating oxidative damage (Munoz and Humeres, 2012); (2) Microglial activation triggering pro-inflammatory cytokine release (e.g., IL-6, TNF-*α*), thereby disrupting synaptic plasticity (Wang et al., 2023); (3) Synergistic interaction with uremic toxins to accelerate β-amyloid deposition. Clinically, the threshold of serum ferritin ≥258.4 μg/L shows promise as an early warning indicator for cognitive decline, with dynamic monitoring enabling timely interventions. Nevertheless, large-scale prospective studies remain imperative to validate these findings.

Previous studies have demonstrated that iron metabolism disorders represent one of the critical mechanisms underlying neurological complications in patients with CKD (Agarwal, 2011). ESRD patients with chronic anemia often require treatment with erythropoietin (EPO) and iron supplementation, which may predispose them to systemic iron overload. Research involving hemodialysis patients has revealed a significant increase in iron deposition in brain regions such as the caudate, which is associated with cognitive decline (Chai et al., 2015). Due to differences in treatment modalities (e.g., continuous solute clearance and hemodynamic stability), the iron metabolism characteristics of PD patients may differ from those observed in hemodialysis patients. Animal studies indicate that the accumulation of uremic toxins can upregulate iron transporters, such as transferrin receptor 1, in the brain, thereby promoting iron deposition across the blood–brain barrier (Deguchi et al., 2006). Nevertheless, the specific pattern of iron deposition in distinct brain regions and its association with cognitive impairment in PD patients remains poorly understood. The present study revealed that the left amygdala and right putamen in PD patients exhibited significantly higher values compared to healthy controls, with the former showing a negative correlation with MoCA scores. These findings suggest that cognitive impairment in peritoneal dialysis patients may be mechanistically linked to iron dysregulation in limbic structures, particularly the amygdala. As a core structure of the limbic system, the abnormal deposition of iron in the amygdala may lead to the generation of free radicals, potentially impairing cognitive function through the following mechanisms. First, excessive iron ions catalyze the production of free radicals, disrupting synaptic plasticity and neurotransmitter balance (e.g., 5-HT and GABA systems), thereby directly affecting emotion regulation and memory consolidation (Thielen et al., 2019). Second, disrupted iron homeostasis may exacerbate anxiety and depressive-like behaviors by modulating the GABAergic neurotransmitter system to enhance aberrant activation of the amygdala-prefrontal neural circuitry, while simultaneously suppressing hippocampal-dependent cognitive processes (Alves et al., 2022; Arora et al., 2024; Zhang et al., 2023).

The precise relationship between peripheral iron metabolism and cerebral iron metabolism has not been fully elucidated. This study also found no significant correlation between magnetic susceptibility values in the left amygdala and levels of hemoglobin, serum iron, ferritin, or transferrin saturation in peritoneal dialysis patients, suggesting that cerebral iron metabolism and systemic iron homeostasis may be regulated through independent mechanisms. First, the integrity of the blood–brain barrier may be compromised in chronic kidney disease, potentially decoupling brain iron deposition from peripheral iron parameters (Faucher et al., 2023). Simultaneously, the expression of key cerebral iron regulatory proteins, such as hepcidin and transferrin receptors, exhibits region-specific patterns within the central nervous system (Zheng and Monnot, 2012). Amygdala-specific iron metabolism pathways may maintain local iron equilibrium via the autophagy-lysosomal system (Xu et al., 2017). Furthermore, neuroinflammation triggered by the accumulation of uremic toxins may activate the iron transport system within microglia, leading to localized iron deposition independent of systemic iron status (Hamed, 2019; Xu et al., 2017).

The present study has several limitations that warrant attention. Firstly, due to the small sample size, we should approach the interpretation and application of our findings with caution. Secondly, the cross-sectional design makes it difficult to establish the causal relationship between iron metabolism indicators and cognitive impairment. In particular, the microinflammatory state commonly existing in peritoneal dialysis patients may simultaneously affect the redistribution of iron metabolism and the permeability of the blood–brain barrier. Longitudinal studies are required to observe the process of iron deposition. Third, systematic measurements of key regulatory factors such as hepcidin and IL-6 were not performed, potentially missing important mediating variables in the inflammation-iron metabolism interaction. In addition, due to the exclusion of non-dialysis CKD patients and HD patients, we cannot clarify the direction of PD’s impact on cerebral iron deposition, nor can we determine if the iron deposition in the left amygdala and right putamen is a PD-specific finding or a common change in ESRD. Meanwhile, we cannot rule out the possibility of similar iron deposition in non-dialysis CKD/HD patients. Cross-sectional studies will be conducted in the future to compare the patterns of cerebral iron deposition among these three groups of patients and longitudinal studies will be carried out to track changes in iron metabolism in non-dialysis CKD patients as their disease progresses, in order to clarify the association between PD and iron abnormalities. Finally, while the AAL template was usually used for brain segmentation, its limitations in defining anatomical boundaries of deep nuclei (e.g., globus pallidus, substantia nigra) may introduce partial volume effect errors.

## 5 Conclusion

In this study, QSM was employed to quantitatively analyze iron deposition changes in the hippocampus, amygdala, caudate nucleus, putamen, globus pallidus, and thalamus. The results demonstrated that the severity of PD-MCI correlates with cerebral iron deposition. Specifically, magnetic susceptibility values in the left amygdala showed a negative correlation with cognitive function assessed by the MoCA. These findings suggest that magnetic susceptibility in the left amygdala, which reflects regional iron deposition, is closely associated with cognitive impairment in PD patients and may hold potential as a candidate imaging indicator for exploring the neuropathological mechanisms of cognitive decline in this population.

## Data Availability

The original contributions presented in the study are included in the article/Supplementary material, further inquiries can be directed to the corresponding authors.
