# Supplementary material for: Regional high iron deposition is linked with cognitive impairments in peritoneal dialysis: a quantitative susceptibility mapping study
Source: Front Aging Neurosci. 2025 Sep 24;17:1660734. doi: 10.3389/fnagi.2025.1660734 (PMC12504288; doi:10.3389/fnagi.2025.1660734)
Supplement: Supplementary file 1 [file Table_1.DOC]

**Supplementary Information**

**Supplementary Table 1** The univariable analysis of the relationship between MoCA scores and clinical indicators in peritoneal patients.

| **Variable** | **β (95%CI)** | ***P* value** |
| --- | --- | --- |
| Age (years) | -0.348 (-0.554, -0.143) | 0.001 |
| Female [n (%)] | -1.896 (-5.593, 1.800) | 0.308 |
| Education (years) | 1.191 (0.485, 1.896) | 0.001 |
| Dialysis period(months) | -0.049 (-0.097, 0.000) | 0.052 |
| BMI (kg/m^2^) | -0.249 (-0.869, 0.371) | 0.424 |
| Diabetes [n (%)] | -0.725 (-5.124, 3.674) | 0.742 |
| Incidence of peritonitis [n (%)] | 0.124 (-3.931, 4.179) | 0.951 |
| Systolic blood pressure (mmHg) | 0.013 (-0.090, 0.116) | 0.805 |
| Diastolic blood pressure (mmHg) | 0.103 (-0.043, 0.250) | 0.163 |
| Laboratory data |  |  |
| White blood cell count (10^9^/L) | 0.158 (-0.638, 0.951) | 0.690 |
| Hemoglobin (g/L) | -0.010 (-0.112, 0.092) | 0.852 |
| Albumin (g/L) | -0.169 (-0.709, 0.371) | 0.533 |
| Alkaline phosphatase (U/L) | -0.011 (-0.034, 0.012) | 0.333 |
| LDH (U/L) | -0.001 (-0.008, 0.006) | 0.725 |
| BUN (mmol/L) | 0.109 (-0.211, 0.430) | 0.495 |
| Uric acid (μmol/L) | 0.003 (-0.019, 0.026) | 0.758 |
| Serum creatinine (umol/L) | 0.002 (-0.005, 0.009) | 0.565 |
| rGFR ml/min/1.73m^2^ | -0.271 (-0.751, 0.209) | 0.262 |
| ***Continued*** | | |
| **Variable** | **β (95%CI)** | ***P* value** |
| Total cholesterol (mmol/L) | -0.317 (-2.148, 1.514) | 0.730 |
| Triglyceride (mmol/L) | -0.542 (-1.861, 0.778) | 0.413 |
| LDL-C | 0.128 (-2.381, 2.637) | 0.919 |
| HDL-C | 0.501 (-5.585, 6.588) | 0.869 |
| iPTH (pg/ml) |  |  |
| Calcium-phosphorus product | -1.350 (-3.217, 0.517) | 0.153 |
| Bicarbonate (mmol/L) | -0.250 (-0.940, 0.441) | 0.471 |
| Serum iron (umol/L) | 0.357 (-0.068, 0.782) | 0.098 |
| Serum ferritin (ug/L) | -0.040 (-0.054, -0.025) | <0.001 |
| Transferrin saturation (%) | -0.310 (-0.567, -0.053) | 0.019 |
| TIBC (umol/L) | -0.133 (-0.343, 0.076) | 0.207 |
| CRP (mg/L) | -0.092 (-0.237, 0.053) | 0.209 |
| D/P Cr at 4h | 2.721 (-14.444, 19.886) | 0.752 |
| Total CCr (L/week) | 0.026 (-0.056, 0.107) | 0.531 |
| Total Kt/V | 0.492 (-1.813, 2.797) | 0.670 |
| Peritoneal function |  |  |
| Low/low average transport (n (%)) | Reference | - |
| High/high average transport (n (%)) | -0.103 (-3.937, 3.732) | 0.957 |

Notes:

BMI: body mass index; LDH: lactate dehydrogenase; BUN: blood urea nitrogen; rGFR: residual glomerular filtration rate; LDL-C: low-density lipoprotein cholesterol; HDL-C: high-density lipoprotein cholesterol; iPTH: intact parathyroid hormone; TIBC: total iron binding capacity; CRP: c-reactive protein; D/P Cr: dialysate/plasma creatinine ratio; CCr: creatinine clearance rete; Kt/V: urea clearance index.

**Supplementary Table 2** Associations between serum ferritin and MoCA scores in different subgroups.

| **Subgroup** | **MoCA (score)** | ***P* for interaction** |
| --- | --- | --- |
|  | **β (95%CI), *P* value** |  |
| Stratified by age |  | 0.818 |
| ≥ 53 years old | -0.034 (-0.050, -0.017), <0.001 |  |
| <53 years old | -0.036 (-0.061, -0.012), 0.005 |  |
| Stratified by gender |  | 0.230 |
| Male | -0.025 (-0.050, -0.001), 0.05 |  |
| female | -0.041 (-0.058, -0.024),<0.001 |  |
| Stratified by BMI |  | 0.105 |
| ≥25 kg/m2 | -0.026 (-0.046, -0.006), 0.014 |  |
| <25 kg/m2 | -0.046 (-0.066, -0.027), <0.001 |  |
| Stratified by education |  | 0.163 |
| ≥8 years | -0.029 (-0.045, -0.014), 0.001 |  |
| <8 years | -0.047 (-0.067, -0.026), <0.001 |  |
| Stratified by Dialysis period |  | 0.083 |
| ≥23 months | -0.052 (-0.075, -0.028), <0.001 |  |
| <23 months | -0.030 (-0.046, -0.013), 0.001 |  |
| Hypertension |  | 0.176 |
| No | -0.082 (-0.162, -0.002), 0.052 |  |
| Yes | -0.035 (-0.049, -0.022), <0.001 |  |
| Diabetes |  | 0.993 |
| No | -0.037 (-0.052, -0.021), <0.001 |  |
| Yes | -0.037 (-0.069, -0.005), 0.030 |  |
| Peritoneal function |  | 0.390 |
| High/high average transport | -0.044 (-0.069, -0.018), 0.002 |  |
| Low/low average transport | -0.033 (-0.049, -0.017), <0.001 |  |

Notes:

Except for the stratification component itself, each stratification factor was adjusted for age, education, dialysis period, and transferrin saturation.

MoCA: montreal cognitive assessment.

**Supplementary Table 3** Correlations between susceptibility of selected brain structures and neuropsychological data.

| **Valiabies** | **Amygdala_L** | | **Putamen_R** | |
| --- | --- | --- | --- | --- |
|  | **r** | ***P*-FDR** | **r** | ***P*-FDR** |
| MoCA (score) | -0.227 | 0.019 | 0.147 | 0.464 |
| SAS (score) | 0.297 | 0.004 | -0.036 | 0.683 |
| SDS (score) | 0.295 | 0.004 | -0.107 | 0.464 |
| SDMT (score) | -0.157 | 0.063 | 0.126 | 0.464 |
| DST-forwards (score) | -0.371 | <0.001 | -0.013 | 0.728 |
| DST-backwards (score) | -0.226 | 0.019 | -0.055 | 0.636 |
| Stroop-word (s) | 0.083 | 0.186 | -0.066 | 0.603 |
| Stroop-color (s) | 0.057 | 0.241 | -0.118 | 0.464 |
| Stroop-interference (s) | 0.158 | 0.062 | -0.106 | 0.464 |
| TMT-A (s) | <0.001 | 0.358 | -0.055 | 0.635 |
| TMT-B (s) | -0.104 | 0.143 | 0.026 | 0.705 |
| VFT  (score) | -0.216 | 0.022 | 0.128 | 0.464 |

Notes:

MoCA: montreal cognitive assessment; SAS: self-rating anxiety scale; SDS: self-rating depression scale;

SDMT: symbol digit modalities test; DST: digit span test; TMT: trail making test; VFT: verbal fluency test

**Supplementary Figure 1** Correlation analysis between the susceptibility of left amygdala and iron metabolism indicators. (A) Hemoglobin; (B) serum iron; (C) serum ferritin; (D) transferrin saturation.
